# Hydrothermal Growth and Orientation of LaFeO_3_ Epitaxial Films

**DOI:** 10.3390/ma17112758

**Published:** 2024-06-05

**Authors:** Guang Xian, Tongxin Zheng, Yaqiu Tao, Zhigang Pan

**Affiliations:** 1 College of Materials Science and Engineering, Nanjing Tech University, Nanjing 211800, China; 202161203216@njtech.edu.cn (G.X.); 202261203296@njtech.edu.cn (T.Z.); taoyaqiu@njtech.edu.cn (Y.T.); 2 State Key Laboratory of Materials-Oriented Chemical Engineering, Nanjing 211800, China

**Keywords:** lanthanum ferrite, hydrothermal synthesis, epitaxial film, orientation

## Abstract

LaFeO_3_ thin films were successfully epitaxially grown on single-crystalline SrTiO_3_ substrates by the one-step hydrothermal method at a temperature of 320 °C in a 10 mol/L KOH aqueous solution using La(NO_3_)_3_ and Fe(NO_3_)_3_ as the raw materials. The growth of the films was consistent with the island growth mode. Scanning electronic microscopy, elemental mapping, and atomic force microscopy demonstrate that the LaFeO_3_ thin films cover the SrTiO_3_ substrate thoroughly. The film subjected to hydrothermal treatment for 4 h exhibits a relatively smooth surface, with an average surface roughness of 10.1 nm. X-ray diffraction in conventional Bragg–Brentano mode shows that the LaFeO_3_ thin films show the same out-of-plane orientation as that of the substrate (i.e., (001)_LaFeO3_||(001)_SrTiO3_). The in-plane orientation of the films was analyzed by φ-scanning, revealing that the orientational relationship is [001]_LaFeO3_||[001]_SrTiO3_. The ω-rocking curve indicates that the prepared LaFeO_3_ films are of high quality with no significant mosaic defects.

## 1. Introduction

Lanthanide perovskite metal oxides have been extensively studied for their unique crystal structure and excellent optical [1], acoustic [2], magnetic [3], and electrical [4] properties. Complex metal oxides with the perovskite structure exhibit greater stability and reliability in terms of high-temperature chemical stability. LnFeO_3_ (Ln is a lanthanide element, such as La, Sm, etc.) is a prototypical perovskite complex metal oxide. Lanthanum ferrite (LaFeO_3_), as a typical rare-earth perovskite complex metal oxide, has found extensive applications in energy storage materials [5], dielectric materials [6], fuel cell electrodes [7], catalytic degradation [8], and gas sensors [9]. LaFeO_3_-related material research has great potential and is expected to provide more stable and sustainable solutions for scientific research and industrial development in many fields [10,11,12,13,14].

LaFeO_3_ thin films are an important class of functional materials because of their structural stability and unique electromagnetic, catalytic, and gas-sensitive properties [15,16,17,18]. A variety of methods, such as pulsed-laser deposition (PLD) [19], molecular-beam epitaxy (MBE) [20], reductive annealing [21], and chemical vapor deposition (CVD) [22], have been applied to prepare epitaxial thin films. Compared to these methods for preparing epitaxial films, the one-step hydrothermal preparation of epitaxial films has the advantages of mild conditions, a lower preparation cost, and a simple procedure, so it is an attractive alternative method to the abovementioned costly techniques [23,24]. The one-step hydrothermal method is also different from the currently common chemical solution deposition (CSD) method, which requires the two-stage treatment of the substrate: a low-temperature pyrolysis (usually 200–450 °C) and a high-temperature sintering (usually 600–850 °C) [25]. The one-step hydrothermal synthesis method for the preparation of epitaxial films involves hydrothermally synthesizing epitaxial films directly on the substrate under certain hydrothermal conditions. The one-step hydrothermal method has been applied to epitaxial films of various types. Huang et al. [26] successfully fabricated a BiFeO_3_ epitaxial film on a SrTiO_3_ substrate via the one-step hydrothermal method, achieving the complete coverage of the substrate at 180 °C for 6 h. The quality of the epitaxial film improved with the increasing temperature and time, and the film exhibited certain dielectric and energy storage properties after annealing. Guo et al. [27] successfully prepared a LaMnO_3_ epitaxial film on a SrTiO_3_ substrate using the one-step hydrothermal method. ADF-STEM cross-section analysis revealed particle exchange and stress strain at the junction of the epitaxial film, confirming expansion outside the lattice plane. Goh et al. [28] successfully grew KNbO_3_ thin films on (100) single-crystal SrTiO_3_ substrates using hydrothermal epitaxy. The KNbO_3_ thin films exhibited an orthogonal structure with the same orientation as the substrate, forming in an island growth pattern before assembling into nano-scale tower structures as the supersaturation decreased. However, there are few articles describing the growth process of epitaxial films on the substrate surface with the increase in the hydrothermal time during the preparation of epitaxial films by hydrothermal methods, and there is no case of LaFeO_3_ epitaxial films prepared by hydrothermal methods. Therefore, in this study, we chose to grow LaFeO_3_ epitaxial films on single-crystal SrTiO_3_ substrates under hydrothermal conditions and investigate their growth process with increasing time to provide a new synthesis method for the subsequent development of LaFeO_3_ epitaxial films.

In this study, we firstly explored the synthesis conditions for the preparation of LaFeO_3_ powders under hydrothermal treatment, and we then successfully prepared LaFeO_3_ epitaxial thin films on SrTiO_3_ single-crystal substrates by the one-step hydrothermal method. The LFO film growth mechanism was studied using a combination of X-ray diffraction (XRD), scanning electron microscopy (SEM), and atomic force microscopy (AFM). The out-of-plane and in-plane orientations of the LaFeO_3_ films were investigated using both conventional X-ray diffraction and high-resolution X-ray diffraction techniques. The quality of the LaFeO_3_ films was assessed via ω-rocking curve measurement, and we investigated the magnetic properties.

## 2. Materials and Method

### 2.1. Synthesis of Bulk LaFeO_3_ Powders

La(NO_3_)_3_·6H_2_O (99.0%, Aladdin Biochemical Technology, Shanghai, China) was used as the lanthanum source, Fe(NO_3_)_3_·9H_2_O (99.0%, Aladdin Biochemical Technology, Shanghai, China) was used as the iron source for the hydrothermal reaction, and KOH (85%, Shanghai Lingfeng Chemical Reagent Co., Ltd., Shanghai, China) was used as the mineralizer. La(NO_3_)_3_·6H_2_O (2.165 g, 0.005 mol) and Fe(NO_3_)_3_·9H_2_O (2.000 g, 0.005 mol) were dissolved in 50 mL of deionized water, the solution turned pale yellow after it had completely dissolved, and then 44.88 g of KOH was added to the above solution in several portions. After the KOH had completely dissolved, the solution changed from pale yellow to reddish brown. Deionized water was added to obtain a suspension mixture of 80 mL and was then transferred to the high hydrothermal reaction (Hastelloy autoclave, 150 mL in volume) for the subsequent hydrothermal treatment. After the hydrothermal reaction was completed and the reaction mixture cooled to room temperature, the solution was centrifuged, and the black precipitate at the bottom of the centrifuge tube was collected and filtered multiple times with deionized water and then dried in an oven at 80 °C for 2 h.

### 2.2. Preparation of LaFeO_3_ Films

**Figure 1:**
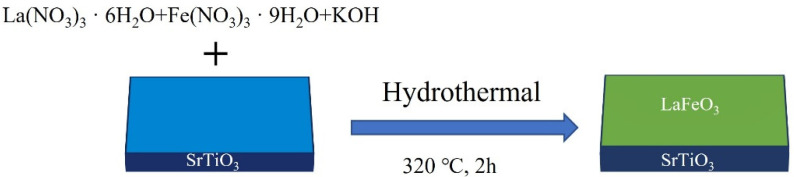
Schematic diagram of the preparation process of LaFeO_3_/SrTiO_3_ epitaxial thin film.

LaFeO_3_ epitaxial films were prepared in a similar way to the preparation of the lanthanum ferrate powder, except that a one-sided polished SrTiO_3_ (001) substrate (5 mm × 5 mm × 0.5 mm) was placed at the bottom of the hydrothermal reactor, as shown in Figure 1, before the transfer of the suspension mixture to the reactor and the prepared films were cleaned using deionized water and ethanol.

### 2.3. Material Characterization

LaFeO_3_ powders and thin films were characterized using a Rigaku SmartLab diffractometer (Rigaku, Tokyo, Japan) with Cu Kα (λ = 1.5418 Å, 40 kV, 100 mA). The powder samples were characterized using the conventional Bragg–Brentano geometry at a scanning speed of 10°/min in the 2θ range of 10–80°, and the thin films were characterized using techniques such as conventional XRD at a scanning speed of 10°/min in the 2θ range of 20–110° and φ-scanning and ω-rocking curves. X-ray photoelectron spectroscopy (XPS) dates were obtained using KRATOS AXIS SUPRA (Shimadzu, Kyoto, Japan). The powder and thin-film samples were characterized by a HitachiTM3000 scanning electron microscope (Hitachi, Tokyo, Japan). The surface roughness of the thin films was characterized by a Bruker Dimension Icon atomic force microscope with tapping type (Bruker, Karlsruhe, Germany).

## 3. Results and Discussion

Before the preparation of the LaFeO_3_ films, a preliminary investigation was conducted to assess the impact of the various hydrothermal reaction parameters, including the temperature, time, mineralizer concentration, and reactant amount, on the synthesis of the LaFeO_3_ powder. Following a series of experiments, it was determined that a hydrothermal reaction temperature of 340 °C for 6 h with a mineralizer concentration of 10 mol/L and a reactant amount of 0.005 mol yielded pure LaFeO_3_ powder. It was also found that temperature had the most significant influence on the purity of the synthesized LaFeO_3_ powder. Consequently, in order to directly observe changes in the composition and morphology with the increasing reaction temperature, temperatures were set at 280 °C, 300 °C, 320 °C, and 340 °C.

**Figure 2:**
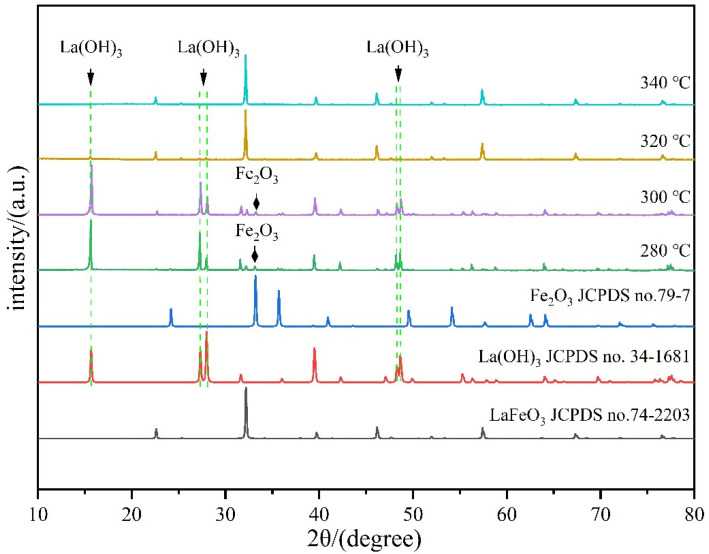
X-ray diffraction patterns of powder samples at different hydrothermal temperatures.

Figure 2 shows the X-ray diffraction patterns of the samples under temperatures of 280 °C, 300 °C, 320 °C, and 340 °C for a duration of 6 h and at a mineralizer concentration of 10 mol/L.

As can be seen from Figure 2, the reaction was incomplete at lower hydrothermal temperatures at 280 °C and 300 °C, and there were three types of substances in the obtained product, LaFeO_3_, La(OH)_3_, and Fe_2_O_3_, with the main substance being La(OH)_3_. With the increase in the reaction temperature to 320 °C, the diffraction peaks of La(OH)_3_ decreased significantly, the diffraction peaks of Fe_2_O_3_ were not visible, and the intensity of the diffraction peaks due to the LaFeO_3_ significantly increased. In the powder diffraction pattern of the product obtained at 340 °C, no reflection peaks due to either La(OH)_3_ or Fe_2_O_3_ were observed, indicating that pure LaFeO_3_ powder was obtained. It was proven that under the same hydrothermal time and mineralizer concentration, the reactants are gradually consumed with the increase in the hydrothermal reaction temperature, so the content of La(OH)_3_ and Fe_2_O_3_ in powder samples is gradually reduced, and the content of LaFeO_3_ is gradually increased. The high diffraction peak intensity and narrow peak with width (HWFM_200_ = 0.14°) indicates that highly crystalline LaFeO_3_ crystals were yielded.

**Figure 3:**
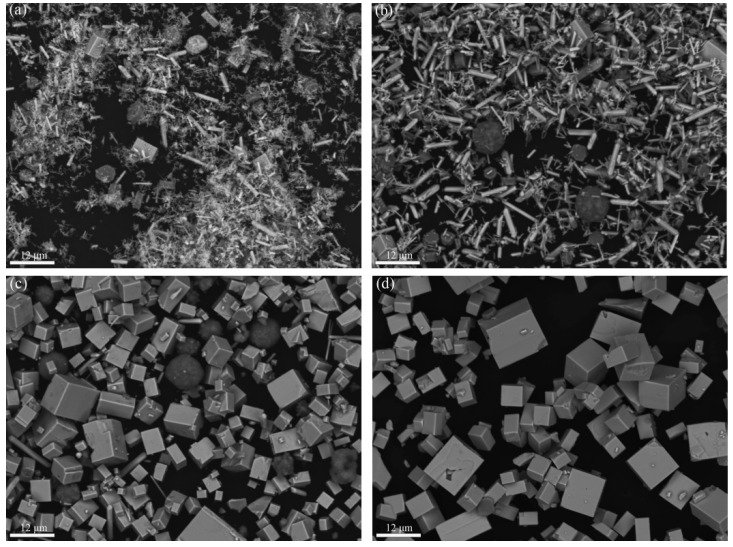
SEM images of powder samples at different reaction temperatures: (**a**) 280 °C, (**b**) 300 °C, (**c**) 320 °C, and (**d**) 340 °C.

Figure 3 shows the SEM images of the powder samples at different reaction temperatures of hydrothermal synthesis.

**Figure 4:**
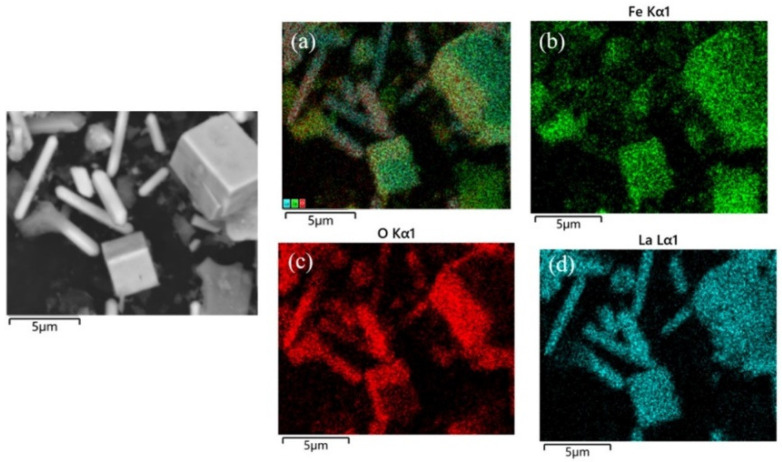
Elemental distribution of cubic and rod-like grains, (**a**) Fe, La, O, (**b**) Fe, (**c**) O, (**d**) La.

**Figure 5:**
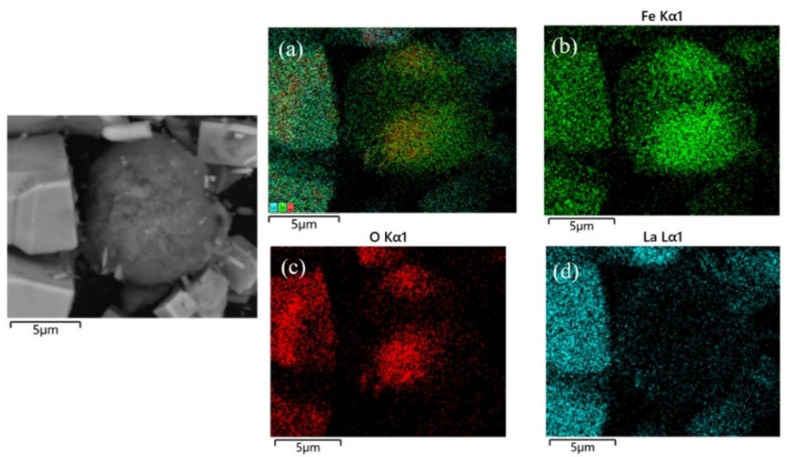
Elemental distribution of spherical grains, (**a**) Fe, La, O, (**b**) Fe, (**c**) O, (**d**) La.

Under 280 °C, the sample mainly consisted of rod-like crystals of different thicknesses, together with a small amount of cubic and spherical grains. As seen from Figure 3a and in comparison with Figure 2, Figure 4, and Figure 5, the rod-like, spherical, and cubic grains are La(OH)_3_, Fe_2_O_3_, and LaFeO_3_, respectively. When the reaction temperature is low, the main substances of the powder sample are La(OH)_3_ and Fe_2_O_3_. As the hydrothermal reaction temperature increases, La(OH)_3_ and Fe_2_O_3_ are gradually consumed and converted into LaFeO_3_ within the same hydrothermal treatment period. At the temperature of 340 °C, only cubic LaFeO_3_ grains are observed in the SEM image shown in Figure 3d. The SEM images are also similar to the experimental results obtained by Tong et al. [29] on the synthesis of LaFeO_3_ by the hydrothermal method.

**Figure 6:**
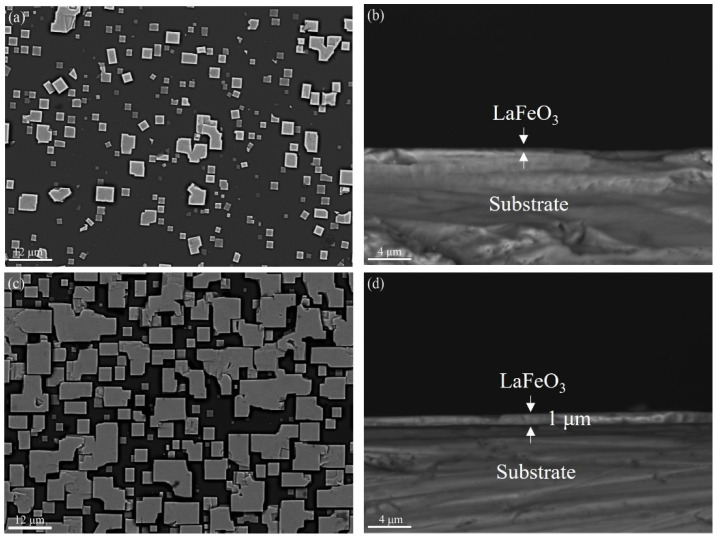
Surface and cross-section SEM images of LaFeO_3_ films at different hydrothermal times: (**a**) 0.5 h sample surface, (**b**) 0.5 h sample cross section, (**c**) 1 h sample surface, and (**d**) 1 h sample cross section.

After the successful preparation of the LaFeO_3_ powders using the one-step hydrothermal method, attempts to grow LaFeO_3_ films on the SrTiO_3_ substrate were made under similar hydrothermal conditions. The LaFeO_3_ films were prepared at a hydrothermal temperature of 320 °C, a mineralizer concentration of 10 mol/L, and various hydrothermal times of 0.5 h, 1 h, 2 h, and 4 h. The morphology changes in the LaFeO_3_ films at different hydrothermal treatment times periods are shown in Figure 6.

**Figure 7:**
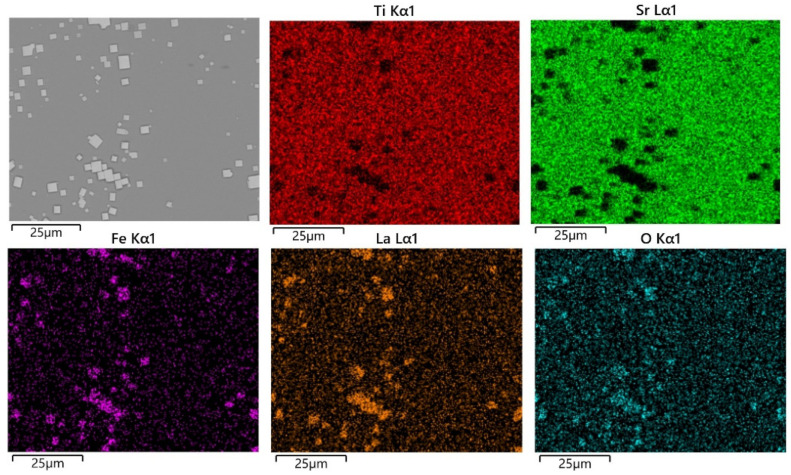
Elemental distribution on the surface of the sample for 0.5 h reaction time.

Figure 6a shows the SEM image of the surface of the SrTiO_3_ substrate with a reaction time of 0.5 h. It can be seen from Figure 7 that the cubes of different sizes in the image are due to LaFeO_3_ grains, and the darker area is due to the SrTiO_3_ substrate. Some adjacent LaFeO_3_ grains were observed to grow fused together. In addition to the low coverage of LaFeO_3_ grains on the SrTiO_3_ substrate at 0.5 h, the grain thickness normal to the substrate was about a few tens of nanometers. When the hydrothermal treatment extended to 1 h, it can be clearly found that the coverage of LaFeO_3_ on the SrTiO_3_ substrate increased significantly, and the thickness of the LaFeO_3_ films was about 1 μm, as shown in Figure 6d.

**Figure 8:**
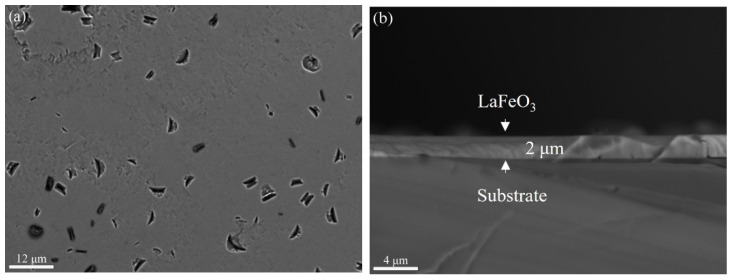
Surface and cross-section SEM images of LaFeO_3_ films on the SrTiO_3_ substrate for 2 h reaction time: (**a**) surface and (**b**) cross section.

**Figure 9:**
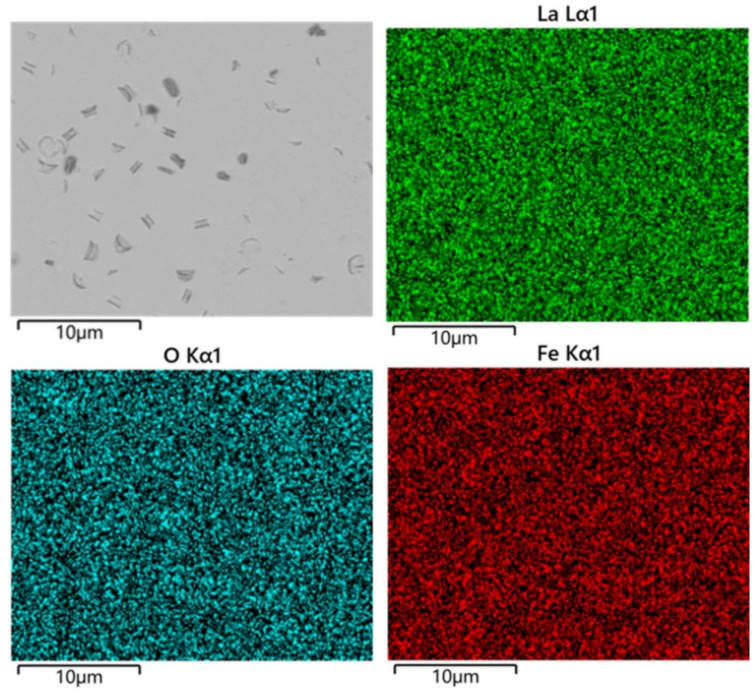
Elemental distribution on the surface of the sample for 2 h reaction time.

From Figure 8a, it can be seen that the SrTiO_3_ substrate is covered thoroughly with LaFeO_3_ films, with dents randomly distributed on the film when the hydrothermal time was increased to 2 h. Elemental mapping of the sample surface at 2 h, as shown in Figure 9, shows that the La, Fe, and O are uniformly distributed throughout the film surface, which is consistent with the SEM image analysis. The thickness of the LaFeO_3_ film obtained at the reaction time of 2 h is about 2 μm, as shown in Figure 8b. The thickness of the films prepared by the hydrothermal method is significantly greater compared to those obtained via other epitaxial methods [19,20,22]. To obtain thinner films using the hydrothermal method, a lower reaction temperature and extension reaction are required.

According to the work by Guo [27] and Ahn [30], as indicated by Figure 6 and Figure 8, the growth mode and growth mechanism of LaFeO_3_ epitaxial thin films grown on SrTiO_3_ substrates by the one-step hydrothermal can be concluded. The growth mode is in accordance with island growth. Its growth process with time can be divided into three growth stages: (Ⅰ) At the initial stage, the SrTiO_3_ substrate is corroded under the conditions of strong alkalinity and high temperature, the cations on the surface are shed to produce active sites, and the free La^3+^ and Fe^3+^ cations in the solution nucleate into ion clusters at the active site (the ion clusters are called “islands”). Due to the short reaction time, the number of “islands” per unit area is limited, the spacing between them is large, and the coverage on the substrate surface is low (Figure 6a, 0.5 h). (Ⅱ) At the intermediate stage, the “islands” that have nucleated exert a significant attraction on the surrounding free ions and the “island” grains spread outward and gradually cover the substrate surface. However, the reaction time is still insufficient for the complete coverage of the substrate surface (Figure 6c, 0.5 h). (Ⅲ) At the final stage, as the “islands” continue to expand around, the “island” and “island” are fused with each other, and the voids between them are filled by LaFeO_3_ grains until the substrate surface is completely covered (Figure 8, 2 h).

**Figure 10:**
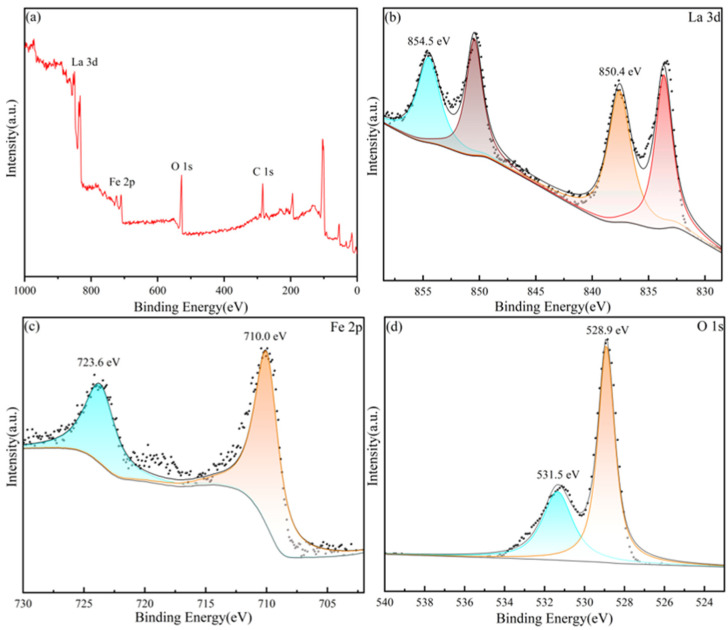
XPS-measured spectra of LaFeO_3_ thin films: (**a**) full spectrum, (**b**) La 3d, (**c**) Fe 2p, and (**d**) O 1s.

XPS was employed to study the chemical state of the metal ions in the LaFeO_3_ films. As can be seen from Figure 10a, only peaks due to La, Fe, O, and C are observed in the full spectrum, which is consistent with the preceding characterizations using XRD and SEM. The two sets of peaks in the La_3d_ spectrum of the LaFeO_3_ film are 854.5 eV and 850.4 eV (corresponding to La_3/2_, high binding energy) and 837.6 eV and 833.7 eV (corresponding to La_5/2_, low binding energy), and the coupling of these two sets of peaks to the La 3d_3/2_ and La 3d_5/2_ spin-orbitals confirms the La^3+^ chemical state. As for the binding energy for iron, the peaks of the Fe 2p_3/2_ and Fe 2p_1/2_ orbitals of the LaFeO_3_ film are located at 710.0 eV and 723.6 eV, respectively, which is in accordance with the binding-energy characteristics of Fe^3+^ [20]. The oxygen signal shown in Figure 6d can be separated into two peaks at 531.5 eV and 528.9 eV, which belong to lattice oxygen ions and adsorbed oxygen, respectively.

**Figure 11:**
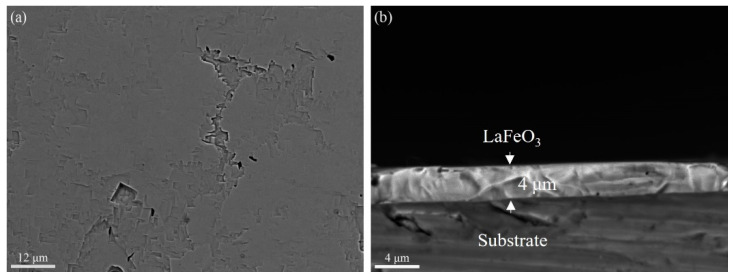
Surface and cross-section SEM images of LaFeO_3_ films on SrTiO_3_ substrate for 4 h reaction time: (**a**) surface and (**b**) cross section.

The extension of the hydrothermal treatment time up to 4 h was carried out to investigate the film surface morphology change. As shown in Figure 11a, the thickness of the film varies for different regions; nevertheless, far fewer dents are observed on the film surface compared with the film grown for 2 h, as shown in Figure 8a. The thickness of the LaFeO_3_ film grown for up to 4 h is about 4 μm.

**Figure 12:**
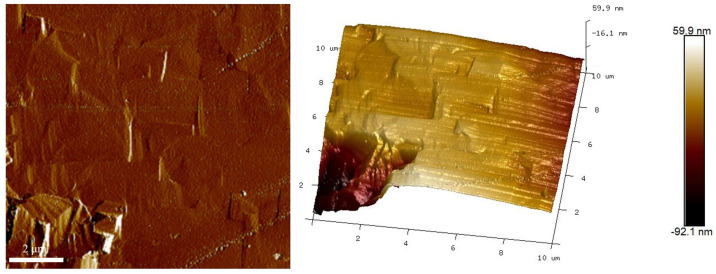
AFM images of LaFeO_3_ films prepared under condition of 4 h reaction time (10 μm × 10 μm).

Figure 12 shows the AFM planar and stereo images of the samples treated for 4 h with a scanning area of 10 μm × 10 μm. It can be seen from Figure 12 that the surface is uniform, and the average roughness (Ra) of the surface was calculated to be 10.1 nm.

**Figure 13:**
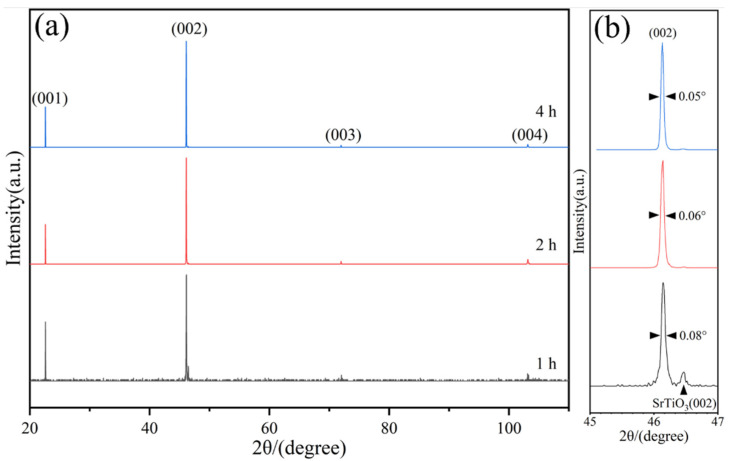
(**a**) Bragg–Brentano X-ray diffraction patterns of LaFeO_3_ thin films prepared at 1 h, 2 h, and 4 h, and (**b**) (002) enlargement of diffraction peaks on the crystal plane.

From Figure 13a, it can be seen that the conventional X-ray diffraction patterns of the LaFeO_3_ thin films prepared at the reaction times of 1 h, 2 h, and 4 h show diffraction peaks at four positions: 2θ = 22.62°, 46.12°, 71.98°, and 103.10°, which correspond to the (001), (002), (003), and (004) crystallographic planes of the LaFeO_3_, respectively. Meanwhile, the diffraction peaks of the SrTiO_3_ substrate can also be found in the X-ray diffraction pattern with a hydrothermal reaction time of 1 h because the substrate surface is not fully covered by the LaFeO_3_ film, as shown in Figure 6c. However, the diffraction peaks of the SrTiO_3_ substrate are not observed in the X-ray diffraction patterns at the reaction times of 2 h and 4 h since the LaFeO_3_ film completely covers the SrTiO_3_ substrate, as indicated by Figure 8a and Figure 11a. Since only (00l) reflections are observed in the conventional X-ray diffraction pattern, the obtained LaFeO_3_ films have a single (001) orientation out of the plane of the film surface. The FWHMs of the HRXRD diffraction peaks on the crystal surface of the LaFeO_3_ film (002) with reaction times of 1 h, 2 h, and 4 h in Figure 13b are 0.08°, 0.06°, and 0.05°, respectively. Compared with LaFeO_3_ films prepared by MBE [20] and PLD [31], the FWHMs are narrower, and the high diffraction peak intensity also proved the films had good quality, high crystallinity, and a low defect density.

**Figure 14:**
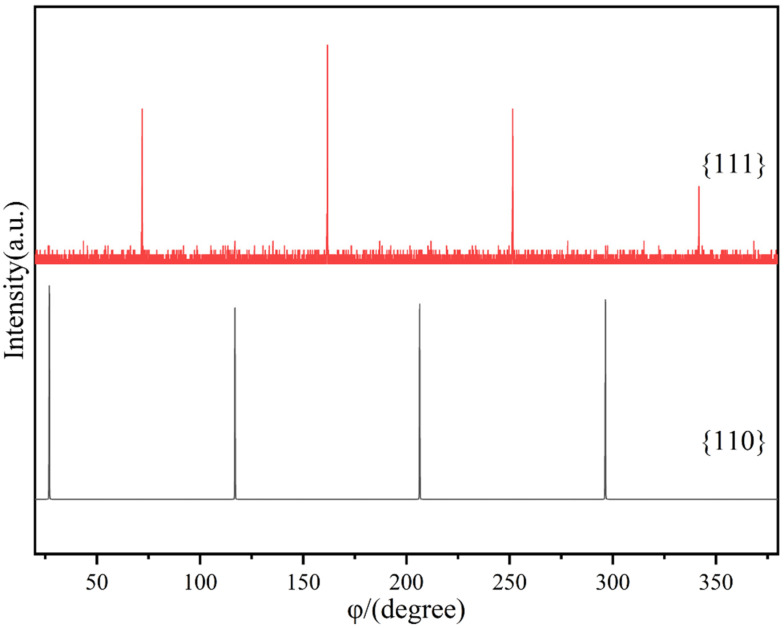
φ-scan diffraction pattern of LaFeO_3_ {111} and {110} crystallographic families at reaction time of 4 h.

While conventional Bragg–Brentano geometry deduced the out-of-plane orientation of the film, from Figure 14, φ-scanning was employed to study the in-plane orientation of the LaFeO_3_ films. The angle between the crystallographic was 54.7°, and between (001) and (110), it was 45°. Prior to the φ-scanning, the sample on the phi-chi cradle was rotated along the chi-axis by the corresponding angle, and then the samples were rotated along the sample surface normal by 360° while the X-ray tube and detector were located at the appropriate position to maintain the corresponding 2θ Bragg angle for the (111) or (110) reflection. Figure 10 shows the φ-scans for the {111} and {110} planes, each of which exhibits four reflections separated by 90°. The φ-scans for the {111} and {110} planes suggest that the LaFeO_3_ films have only one in-plane orientation. In Figure 10, the signal to noise of the {111} reflections is significantly worse than that of the {110} reflections due to the relative low reflection intensity. The diffraction intensity of (110) is about 8.2 times that of (111).

Conventional Bragg–Brentano diffraction and φ-scanning suggest that the LaFeO_3_ films have the same out-of-plane and in-plane orientations as those of the SrTiO_3_ substrate. Therefore, the LaFeO_3_ films were epitaxially grown on the SrTiO_3_ substrate with the orientation relationship (001)_LaFeO3_||(001)_SrTiO3_ and [001]_LaFeO3_||[001]_SrTiO3_.

**Figure 15:**
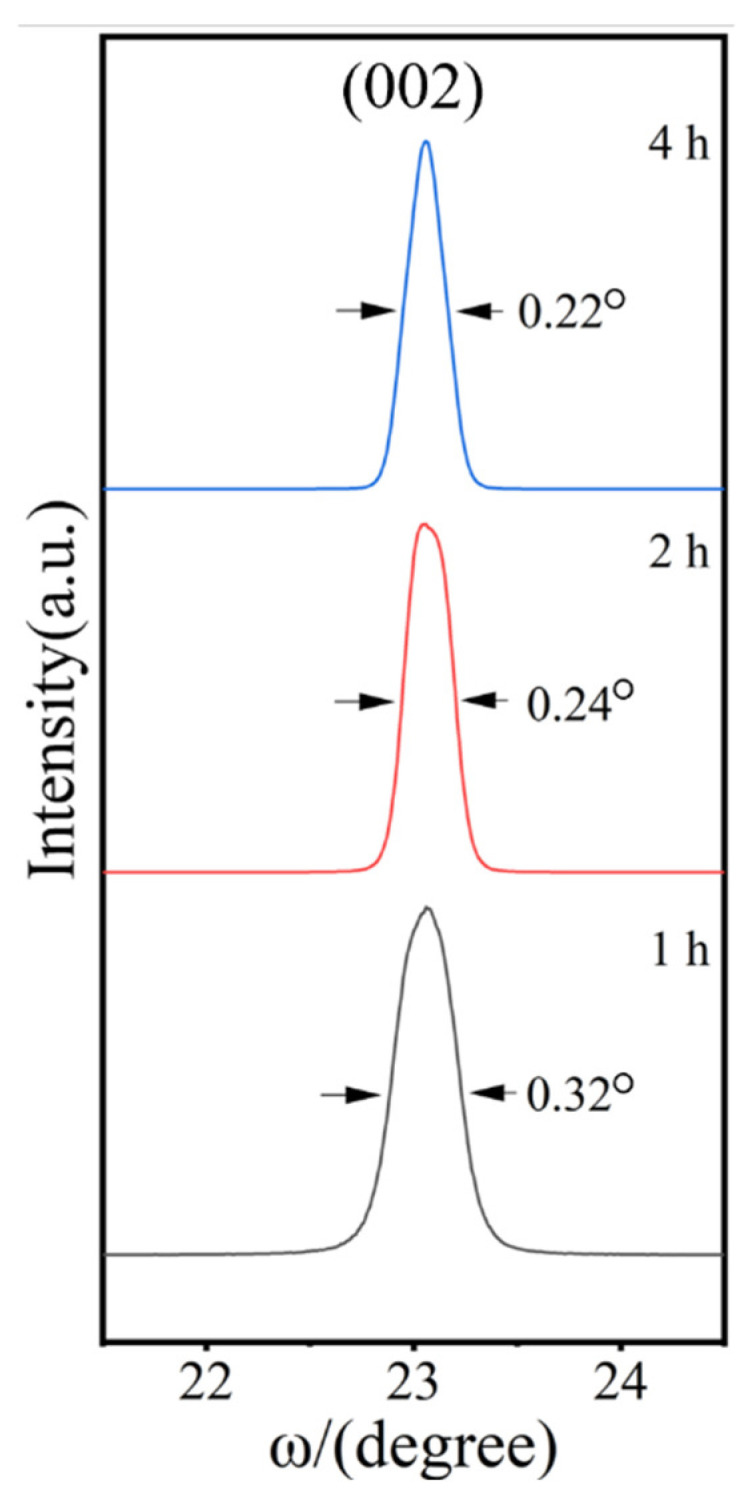
Rocking curves (ω-scans) of LaFeO_3_ (002) crystal surface at reaction time durations of 1 h, 2 h, and 4 h.

Figure 15 shows the rocking curves of the LaFeO_3_ films at the reaction time durations of 1 h, 2 h, and 4 h. The symmetric and narrow rocking curve shown in Figure 15 indicates the high quality of the LaFeO_3_ films. Furthermore, the FWHM of the rocking curve decreases as the hydrothermal treatment time duration increases, indicating that there were less film mosaic defects with the increase in the film thickness.

**Figure 16:**
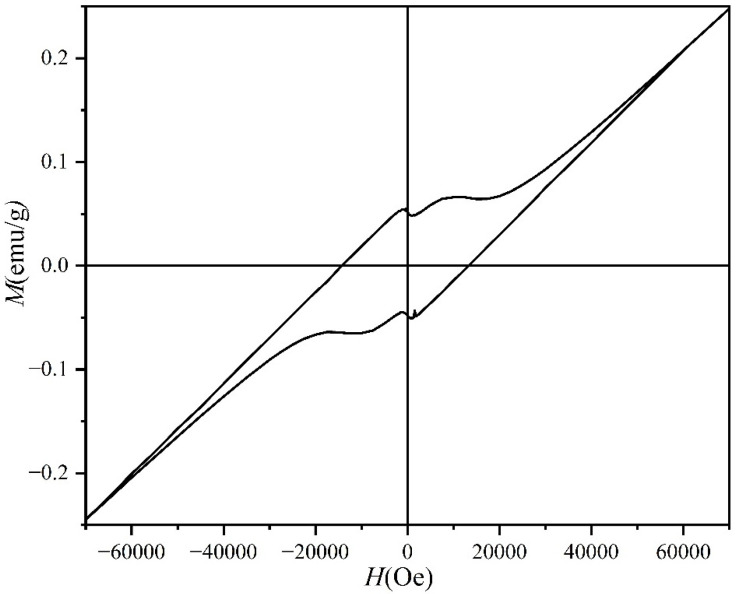
Magnetization hysteresis curves at room temperature for LaFeO_3_ thin film at a hydrothermal time of 4 h.

At the end, we explored the magnetic properties of the LaFeO_3_ thin films at a hydrothermal reaction time of 4 h. Figure 16 shows the magnetic hysteresis loop at room temperature. The results show that the LaFeO_3_ films exhibit some magnetic properties at room temperature, indicating their ferromagnetic order. The coercivity is 12,500 Oe and the residual magnetization strength is 0.054 emu/g. According to the available studies, the ferromagnetic behavior of LaFeO_3_ thin film is due to the uncompensated spins at the surface and the canted internal spin by the tilt of FeO_6_ octahedral units [32,33].

## 4. Conclusions

Pure LaFeO_3_ powder was successfully prepared via the one-step hydrothermal method at 340 °C for 6 h under 10 mol/L KOH. Based on the hydrothermal preparation of LFO powder, LaFeO_3_ epitaxial films were then obtained via the one-step hydrothermal method at 320 °C for 4 h under 10 mol/L KOH, and the average roughness (Ra) of the surface was calculated to be 10.1 nm. The growth mode of the epitaxial film was island growth, and the effect of the hydrothermal reaction time on the hydrothermal synthesis of the LFO films was studied. The extended growth process during the hydrothermal reaction can be summarized as follows: Under high-temperature hydrothermal conditions and strong alkalinity, the substrate is etched to produce active sites. Free La^3+^ and Fe^3+^ in the solution deposited and nucleated at these active sites, forming LaFeO_3_ ion clusters. These ion clusters continued to grow, gradually increasing in size and merging with each other, ultimately forming a LaFeO_3_ epitaxial film. The out-of-plane orientation of the LaFeO_3_ films was analyzed using conventional diffraction. The in-plane orientation of the LaFeO_3_ films was analyzed using the φ-scan diffraction technique. The orientation analysis of the LaFeO_3_ films suggests that LaFeO_3_ was epitaxially grown on the SrTiO_3_ substrate with the orientation relationship (001)_LaFeO3_||(001)_SrTiO3_ and [001]_LaFeO3_||[001]_SrTiO3_. The high quality of the film is confirmed by the rocking curves, and there were fewer film mosaic defects with the increase in the film thickness. And the LaFeO_3_ thin film obtained displayed a ferromagnetic hysteresis loop, with a coercivity of 12,500 Oe and a remnant magnetization of 0.054 emu/g obtained at room temperature.

## Data Availability

The original contributions presented in the study are included in the article, further inquiries can be directed to the corresponding author.
